# Quantum Chemical Investigation into the Structural Analysis and Calculated Raman Spectra of Amylose Modeled with Linked Glucose Molecules

**DOI:** 10.3390/molecules29122842

**Published:** 2024-06-14

**Authors:** Dapeng Zhang, Naoki Kishimoto

**Affiliations:** Department of Chemistry, Graduate School of Science, Tohoku University, 6-3, Aoba, Aramaki, Aoba-ku, Sendai 980-8578, Japan; zhang.dapeng.c5@tohoku.ac.jp

**Keywords:** amylose, calculated Raman spectra, glucose linkage, quantum chemical calculation, structural analysis

## Abstract

This study presents a quantum chemical investigation into the structural analysis and calculated Raman spectra of modeled amylose with varying units of linked glucose molecules. We systematically examined the rotation of hydroxymethyl groups and intramolecular hydrogen bonds within these amylose models. Our study found that as the number of linked glucose units increases, the linear structure becomes more complex, resulting in curled, cyclic, or helical structures facilitated by establishing various intramolecular interactions. The hydroxymethyl groups were confirmed to form interactions with oxygen atoms and with hydroxymethyl and hydroxyl groups from adjacent rings in the molecular structures. We identified distinct peaks and selected specific bands applicable in various analytical contexts by comparing their calculated Raman spectra. Representative vibrational modes within selected regions were identified across the different lengths of amylose models, serving as characteristic signatures for linear and more coiled structural conformations. Our findings contribute to a deeper understanding of amylose structures and spectroscopic signatures, with implications for theoretical studies and potential applications. This work provides valuable reference points for the detailed assignment of Raman peaks of amylose structure, facilitating their application in broader research on carbohydrate structures and their associated spectroscopic properties.

## 1. Introduction

Starch, a ubiquitous component of carbohydrate-rich foods, serves as a primary energy source for human metabolism [1,2,3,4,5] and holds considerable promise for the development of biopolymer composite materials [6,7,8], such as bioplastics [9,10]. Offering a sustainable alternative to conventional petroleum-based plastics, these bioplastics thereby contribute to the mitigation of environmental concerns associated with non-biodegradable synthetic polymers [11,12,13]. As an abundant and biodegradable polysaccharide endowed with a chemically modifiable structure, starch has attracted substantial attention as a promising raw resource for advanced functional materials [14]. However, further exploration and exploitation of starch necessitates a comprehensive elucidation of its intricate structural features across multiple length scales, particularly those of its amylose and amylopectin components. Amylose is a linear polysaccharide composed of α-1,4 glycosidic linkages of glucose units, forming a helical structure stabilized by intramolecular hydrogen bonds.

In contrast, amylopectin features a branched structure, where the linear glucose backbone is interconnected by α-1,4 glycosidic linkages interconnected with additional α-1,6 glycosidic points [15]. The relative proportions of amylose and amylopectin in starch vary, and their distinct assembled features contribute to the potential for forming diverse structures, thereby rendering the structural composition of starch replete with various conformations arising from the interplay between these components, enabling the formation of intricate polymerized architectures [16,17,18]. Given the pivotal role of α-1,4 glycosidic linkages in amylose and amylopectin, a comprehensive structural analysis of the linear glucose linkages becomes imperative. Elucidating the intricate details of these structural features across multiple length scales will facilitate the understanding and development of tailor-made starch-based materials with customized functionalities, unlocking their potential for various applications across diverse domains.

Raman spectroscopy is a versatile and powerful analytical technique for investigating structural information and intramolecular interactions of carbohydrate polymers [19]. This technique can be complemented by quantum chemical calculations, which enable the examination and detailed assignment of the observed Raman spectra of compounds, including amylose and amylopectin [20,21]. In this study, we perform quantum chemical calculations to investigate the structural features of linear glucose models and their corresponding calculated Raman spectra. By combining conformational analysis and assignment of the calculated Raman spectra, we aim to explain the structural details of amylose and amylopectin, thereby facilitating the understanding of their polymerization properties and potential bio-inspired applications.

## 2. Calculation Details

The investigation commenced with the initial optimization of amylose models, consisting of four glucose units (4Glc), utilizing the B3LYP/3-21G level of theory. Uniform orientation of two out of four hydroxymethyl groups was presumed throughout the calculations, performed using the GRRM (Global Reaction Route Mapping) program [22,23] interfaced with Gaussian 16 [24]. Subsequent refinement encompassed further optimization and Raman spectra calculations at the CAM-B3LYP/6-311+G(d,p) level of theory [25,26,27,28,29] using Gaussian 16. To address potential conformational influences on the Raman spectra, a conformational exploration was conducted employing the PM6 semi-empirical method [30] implemented through the ADDF algorithm [31,32,33,34] within the GRRM software (GRRM 17). This algorithm was applied exclusively to the ten lowest local minima (NLowest = 10). A constrained exploration of local minima was performed to minimize computational costs by bypassing transition state optimizations. Tracking the seven largest anharmonic downward distortions (ADDs) along the reaction coordinates of the computed equilibrium (EQ) structures (l-ADDF7) was conducted. The NoBondRearrange option was employed to expedite the search for structures without bond rearrangements. Subsequently, the ten most stable structures identified in the conformational search were optimized along with their corresponding Raman spectra at the CAM-B3LYP/6-311+G(d,p) level of theory using Gaussian 16. Additionally, the amylose structures comprising two, six, and eight glucose units (2Glc, 6Glc, and 8Glc) were investigated for comparison, beginning with initial optimization via the B3LYP/3-21G method utilizing the GRRM program, followed by successive optimization and Raman spectra calculation employing the CAM-B3LYP/6-311+G(d,p) level of theory for 2Glc and 6Glc, while for 8Glc the CAM-B3LYP/6-31G(d) approach [35,36,37,38,39,40,41,42,43,44] within Gaussian 16. The dihedral angles related to the hydroxymethyl groups of each glucose ring were manually adjusted among the 2Glc, 4Glc, 6Glc, and 8Glc models to analyze the formation of intramolecular hydrogen bonds.

Given the potential for intramolecular interactions between hydroxymethyl and hydroxy groups to induce coiling of the linear structure, we additionally examined the conformational landscape without imposing manual selection of molecular dihedral angles. Instead, the ADDF algorithm generated a diverse ensemble of potential structural configurations. This systematic computational algorithm explored various molecular structures, enabling the automatic generation of plausible models while minimizing potential biases that could arise from manual adjustments. The ADDF calculation was initiated from an optimized 4Glc model, wherein the ten lowest local minima (NLowest *=* 10) and only the equilibrium (EQ) structures were traced. In order to achieve computational efficiency, the seven largest ADDs (l-ADD7) surrounding each stable minima point were followed. The conformational exploration was conducted using the semi-empirical PM6 method, yielding 169 stable structures. The ten most stable structures found in the exploration were re-optimized at the CAM-B3LYP/6-311+G(d,p) level of theory, and their Raman spectra were calculated at the same level.

## 3. Results and Discussion

### 3.1. Structural Analysis and Calculated Raman Spectra of 4Glc

**Figure 1:**
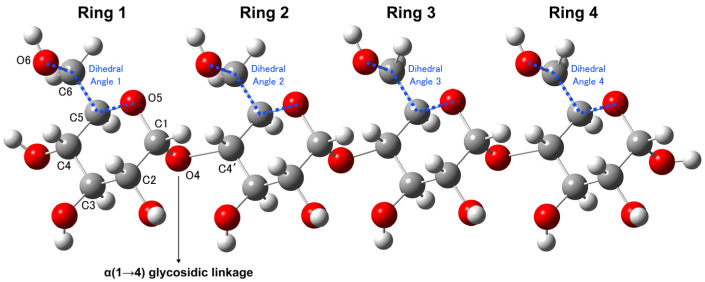
Atom labeling and the dihedral angles examined within four rings in a 4Glc structure. Red balls represent O atoms, white balls represent H atoms, and gray balls represent C atoms. The remaining figures are consistent with the representation.

**Figure 2:**
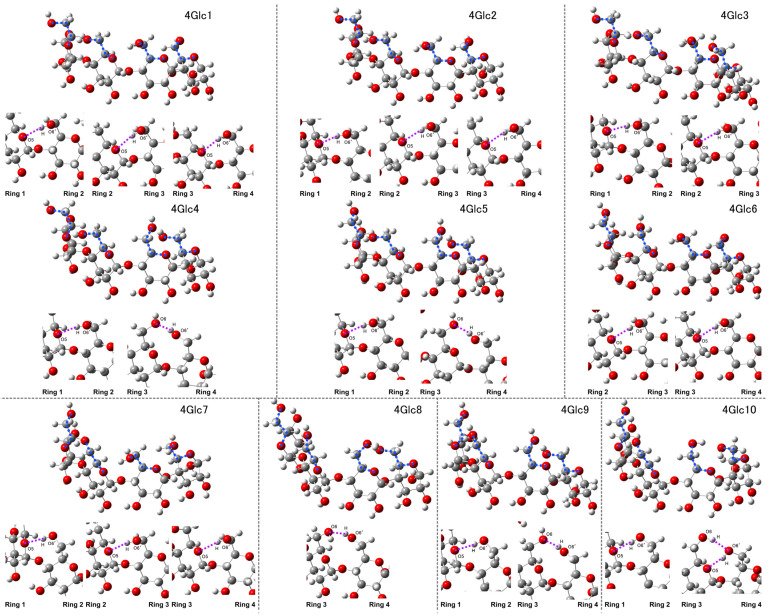
Structural changes observed in 4Glc1-10 models, with dihedral angles highlighted by blue dashed lines and weak intramolecular hydrogen bonds denoted by purple dashed lines.

**Figure 3:**
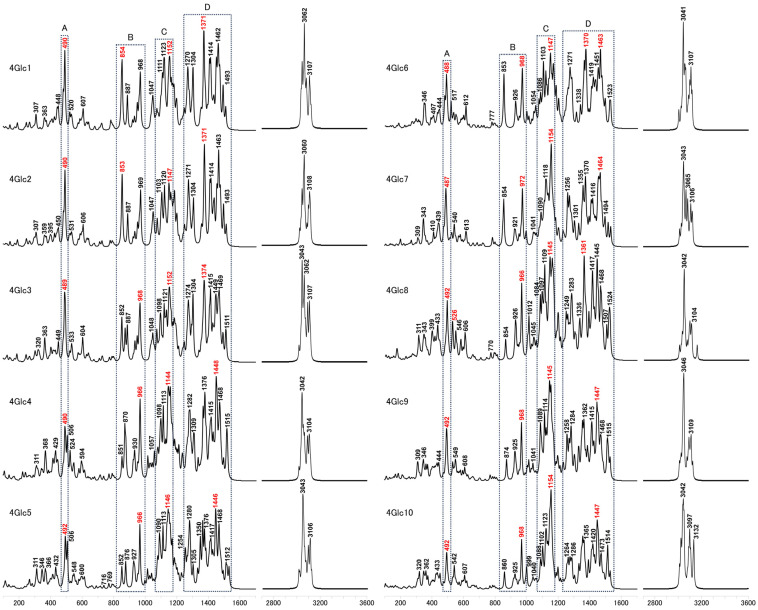
Calculated Raman spectra for 4Glc1-10 models, highlighting bands with the highest intensity in four delineated regions, A, B, C, and D, denoted in red.

**Figure 4:**
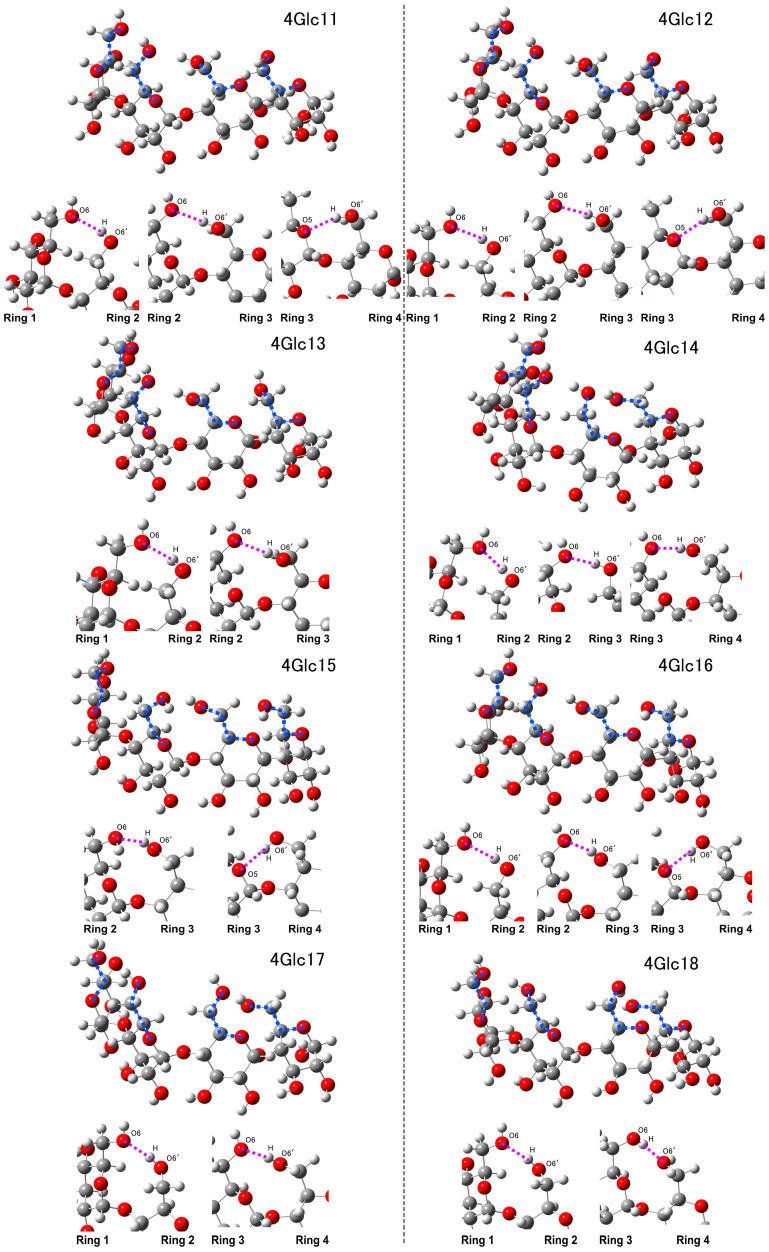
Structural changes observed in 4Glc11-18 models, with dihedral angles highlighted by blue dashed lines and weak intramolecular hydrogen bonds denoted by purple dashed lines.

**Figure 5:**
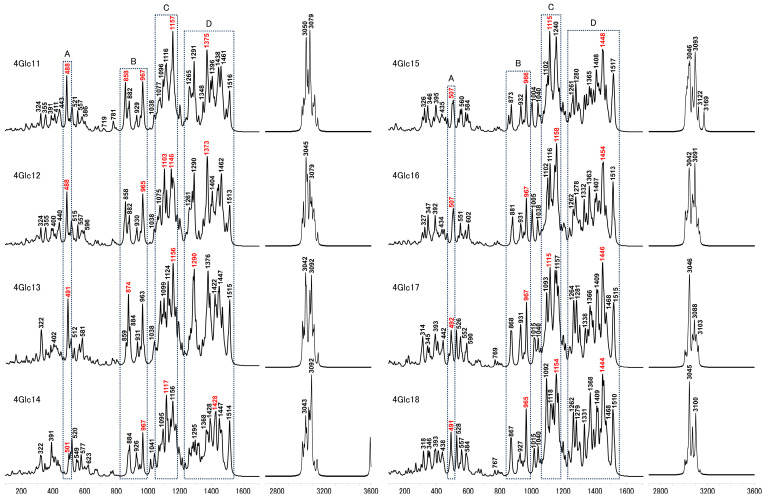
Calculated Raman spectra for 4Glc11-18 models, highlighting bands with the highest intensity in four delineated regions*,* A, B, C, and D, denoted in red.

The initial models were constructed by manipulating the dihedral angles between the O5 and C5 atoms within the ring, as well as those between the C6 and O6 atoms of hydroxymethyl groups linked to the ring (O5-C5-C6-O6), as shown in Figure 1. Detailed parameters governing these adjustments are outlined in Supporting Information, Tables S1 and S2. Subsequent structural optimization resulted in the convergence of certain structures toward similar conformations, which can be attributed to the interplay of hydroxymethyl groups forming intramolecular hydrogen bonds, as the structures depicted in Figure 2 (with calculated Raman spectra in Figure 3) and Figure 4 (with calculated Raman spectra in Figure 5), and Table 1 and Table 2. Two distinct types of intramolecular hydrogen bonds are confirmed: O5⋯H-O6′, formed between a ring and the hydroxymethyl groups at the succeeding ring, and O6⋯H-O6′, formed between the hydroxymethyl groups of two adjacent rings. Notably, O*m*⋯H-O*n*′ indicates that the hydrogen bonds were formed between the O*m* atom of one ring and the H-O*n*′ group from another nearby ring, where the apostrophe (′) denotes a different glucose ring. While these interactions are comparatively weaker than those established between amylose during polymerization, they engender diverse conformational arrangements within the molecular framework. 

4Glc1 and 4Glc2 show analogous conformations, characterized by the presence of three intramolecular hydrogen bonds (O5⋯H-O6′ type) linking ring 1 to ring 2, ring 2 to ring 3, and ring 3 to ring 4. These weak interactions exhibit bond angles of approximately 158.21–161.46 degrees and lengths spanning 2.05–2.10 angstroms. The hydroxymethyl group of ring 4 in 4Glc3 exhibited a different rotational conformation compared to that in 4Glc1 and 4Glc2, with a dihedral angle of 78.30 degrees instead of around −81 to −82 degrees. This variance resulted in the disruption of the hydrogen bond between rings 3 and 4. Additionally, intramolecular hydrogen bonds (O5⋯H-O6′ type) were observed between rings 1 and 2, as well as between rings 2 and 3. 4Glc4 and 4Glc 5 exhibit similar intramolecular interactions, with hydrogen bonds of the O5⋯H-O6′ type observed between ring 1 and ring 2, while another type (O6⋯H-O6′) is confirmed between ring 3 and ring 4. This result is accompanied by the disruption of hydrogen bonds between ring 2 and ring 3. Interestingly, the dihedral angle 1 for 4Glc4 is −82.18 degrees, while that of 4Glc5 is −177.38 degrees, indicating significant differences in the conformational rotation of hydroxymethyl groups at ring 1. 

4Glc6 exhibits unique weak interactions, characterized by two hydrogen bonds of the O5⋯H-O6′ type, between ring 2 and ring 3, as well as between ring 3 and ring 4. The presence of three hydrogen bonds (O5⋯H-O6′ type) across rings 1 to 2, 2 to 3, and 3 to 4 in 4Glc1-2 was also observed in 4Glc7. Nonetheless, notable conformational disparities were identified in dihedral angle 1 (−66.70 and −66.67 degrees for 4Glc1-2, −174.49 degrees for 4Glc7) and dihedral angle 2 (−81.12 and −81.09 degrees for 4Glc1-2, 168.55 degrees for 4Glc7). In 4Glc8, we observed a single hydrogen bond of the O6⋯H-O6′ type, formed between ring 3 and ring 4, with bond angles of 158.83 degrees and a bond length of 1.98 angstroms. The dihedral angles 1 and 2 exhibited similar values in 4Glc9 and 4Glc10, measuring −174.92 and −173.37 degrees and 168.24 and 170.75 degrees, respectively. In both 4Glc9 and 4Glc10, an O5⋯H-O6′ type hydrogen bond was observed between ring 1 and ring 2, while no hydrogen bond interaction was observed between ring 2 and ring 3. Although both structures exhibited an O6⋯H-O6′ type hydrogen bond between ring 3 and ring 4, the difference in dihedral angles 3 (72.53 and 94.97 degrees for 4Glc9 and 4Glc10, respectively) and dihedral angles 4 (148.49 and 171.18 degrees for 4Glc9 and 4Glc10, respectively) resulted in the simultaneous presence of an O5⋯H-O6′ type hydrogen bond between ring 3 and ring 4 in 4Glc10.

The Raman spectra calculated for 4Glc1-10 exhibited noticeable disparities in relative intensity and subtle shifts in band positions across the spectrum. Notably, relative intensity comparisons were conducted within each designated molecule region rather than between different structures. These findings are shown in Figure 3. It is important to note that all the calculated results presented in this work are from the Raman activity spectrum. Our analysis specifically targeted four distinct regions to elucidate the conformational characteristics in depth. The calculated Raman spectra of 4Glc1 and 4Glc2 exhibit similarities attributable to their shared conformations. However, discernible disparities were observed, particularly in 4Glc2, wherein a distinct band emerged at approximately 1103 cm^−1^ within region C, alongside an intensified band at 1304 cm^−1^ within region D. By contrast, 4Glc3 displayed significant alternations, particularly characterized by a diminished relative intensity at 852 cm^−1^ within region 2, a reduction in relative intensity of the 1121 cm^−1^ band within region 3, as well as attenuated signals at 1374 and 1469 cm^−1^ within region 4, in addition to the attenuation observed at 1152 cm^−1^ within region 4. 4Glc4 and 4Glc5 exhibited a novel peak around 506 cm^−1^ within region 1, while 4Glc4 displayed notably increased intensities at the 870 cm^−1^ band within region 2, as well as at the 1309 and 1515 cm^−1^ bands within region 4. Moreover, an increased intensity was observed around the 1446 or 1448 cm^−1^ bands in region 4, for both 4Glc4 and 4Glc5. 

Although 4Glc6 and 4Glc7 share similar parameters of dihedral angles 1, 3, and 4, the latter exhibited a hydrogen bond (O5⋯H-O6′ type) between ring 1 and ring 2, as depicted in Figure 2. The calculated Raman spectra of both models are nearly identical in regions 1 and 2. However, in region C, the intensity of the 1154 cm^−1^ peak in 4Glc7 significantly dominated. In contrast, in 4Glc6, the intensity differences between the most intensified band of 1147 cm^−1^ within region C and the two strong peaks (1370 and 1463 cm^−1^) in region D are less pronounced. In the B region of 4Glc8-10, the bands exhibited decreased intensities at 854, 874, and 860 cm^−1^, respectively. In the C region of 4Glc8, the peak intensities at 1109 and 1145 cm^−1^ are similar, whereas, in 4Glc9 and 4Glc10, only the band at 1145 or 1154 cm^−1^ showed a prominent peak. Furthermore, the strongest peak in the C region of 4Glc8 (1145 cm^−1^) is comparable in intensity to the strongest peak in the D region (1361 cm^−1^), whereas, in 4Glc9 and 4Glc10, the intensity of this peak notably decreased. Additionally, the intensity of the 1286 cm^−1^ bands in 4Glc10 decreased compared to 4Glc9, and the difference in intensity between the 1286 cm^−1^ and 1264 cm^−1^ bands is less pronounced. These three structures exhibited prominent peaks at 1524, 1515, and 1514 cm^−1^, similar to the independent sharp peak observed at 1515 cm^−1^ in 4Glc4.

The conversion of dihedral angles 1 and 2 to positive values resulted in distinct conformations within the interconnected four-glucose molecules, as illustrated in Figure 4. Both 4Glc11 and 4Glc12 exhibited akin structures characterized by three intramolecular hydrogen bonds. Specifically, hydrogen bonds of the O6⋯H-O6′ type were established between the hydroxymethyl groups of ring 1 and ring 2 and between ring 2 and ring 3. Concurrently, the O5⋯H-O6′ type was observed to form between ring 3 and ring 4. In contrast, a significant deviation in the dihedral angle 4 of 4Glc13 was observed, measuring 77.92 degrees, in contrast to those of 4Glc11 and 4Glc12 (−81.33 and −82.14 degrees, respectively). This alteration resulted in the hydroxymethyl group in ring 4 relocating farther from ring 3, disrupting the intermolecular interactions with ring 3. Notably, only two hydrogen bonds of the O6⋯H-O6′ type were evident, occurring between ring 1 and ring 2, as well as between ring 2 and ring 3, respectively. In 4Glc14, three intramolecular hydrogen bonds of the O6⋯H-O6′ type were identified, attributed to rotations of hydroxymethyl groups between ring 1 and ring 2, between ring 2 and ring 3, and between ring 3 and ring 4, respectively. 4Glc15 and 4Glc16 displayed similarity in dihedral angles 1, 3, and 4, while a notable disparity arose in dihedral angle 2, exhibiting values of 57.27 and 96.93 degrees, respectively. This discrepancy implied that conformational variations primarily stem from the rotational dynamics of the hydroxymethyl group within ring 2. Notably, 4Glc15 manifested intramolecular hydrogen bonds formed between ring 2 and ring 3 (O6⋯H-O6′ type) and between ring 3 and ring 4 (O5⋯H-O6′ type). Additionally, in 4Glc16, alongside these aforementioned interactions, the hydroxymethyl groups within ring 1 and ring 2 facilitated the formation of weak bonds of the O6⋯H-O6′ type. In both 4Glc 17 and 4Glc 18, analogous interactions between the hydroxymethyl groups within rings 1 and 2, as well as between rings 3 and 4, led to the formation of O6⋯H-O6′ type hydrogen bonds. However, due to differences in dihedral angles 2 and 3, with values of 139.65 and 73.00 degrees, respectively, for the former structure and 151.75 and 64.18 degrees, respectively, for the latter, these molecules failed to establish interactions between rings 2 and 3.

Conformational similarities between 4Glc11 and 4Glc12 were confirmed, complemented by comparable calculated Raman spectra in regions A and B, as shown in Figure 5. However, in the case of 4Glc11, the dominance of the 1157 cm^−1^ peaks in region C exceeded even the most prominent band in region D (1375 cm^−1^). In addition, region C of 4Glc11 showed two notable bands at 1103 and 1146 cm^−1^, albeit with relative intensities lower than the predominant band within region D (1373 cm^−1^). Conversely, 4Glc12 experienced considerable alternations in dihedral angle 4, as indicated by a notable amplification of the 874 cm^−1^ band in its region B, surpassing the intensity of the 963 cm^−1^ bands. Notably, the 1156 cm^−1^ band in region C of 4Glc13 mirrored the behavior in 4Glc11. Additionally, region D of 4Glc12 featured two strong peaks at 1290 and 1376 cm^−1^, accompanied by a significant increase in the intensity of the 1515 cm^−1^ band. In region A of 4Glc14, the peak at 501 cm^−1^ exhibited relatively low intensity, while region B shared similarity with the corresponding regions of 4Glc15 and 4Glc16. Within region C, two peaks were discerned at 1117 and 1156 cm^−1^, mirroring the spectral characteristics observed in 4Glc15 (which manifested peaks at 1115 and 1240 cm^−1^). In contrast, in 4Glc16, a solitary dominant peak appeared at 1158 cm^−1^ within the same spectral region. Contrasted with 4Glc14, regions D of 4Glc15 and 4Glc16 showcased robust peaks aligning with the 1448 or 1454 cm^−1^ bands. Region C of 4Glc17 revealed the presence of three discernible peaks at 868, 931, and 967 cm^−1^, with escalating relative intensities. By contrast, the intensity of the 927 cm^−1^ band in 4Glc18 is noticeably diminished compared to those at 867 and 965 cm^−1^. Additionally, distinctions between the two compounds emerged in region C, wherein the 1093 cm^−1^ peak exhibited reduced intensity relative to the 1115 cm^−1^ peak in 4Glc17, while conversely observed in 4Glc18 (1092 to 1118 cm^−1^ bands). Additionally, an augmentation in the intensity of the 1368 cm^−1^ peak in region D became evident in 4Glc18.

**Figure 6:**
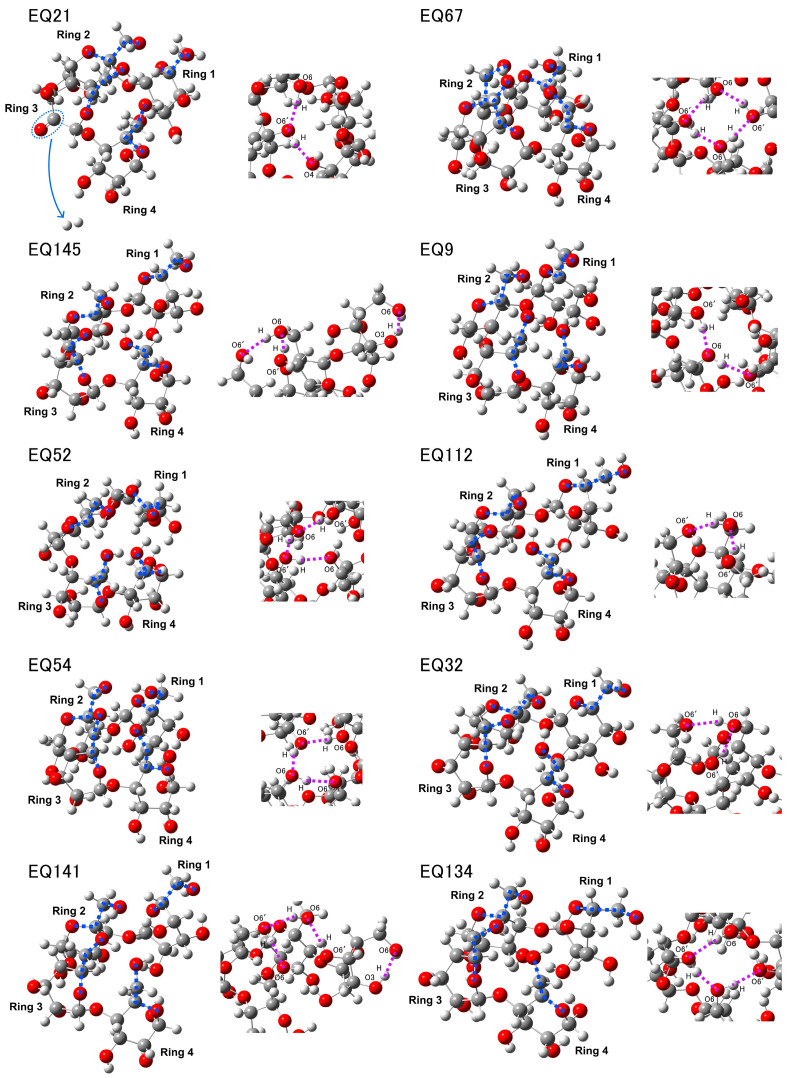
Structural changes observed in explored models, with dihedral angles highlighted by blue dashed lines and weak intramolecular hydrogen bonds denoted by purple dashed lines.

**Figure 7:**
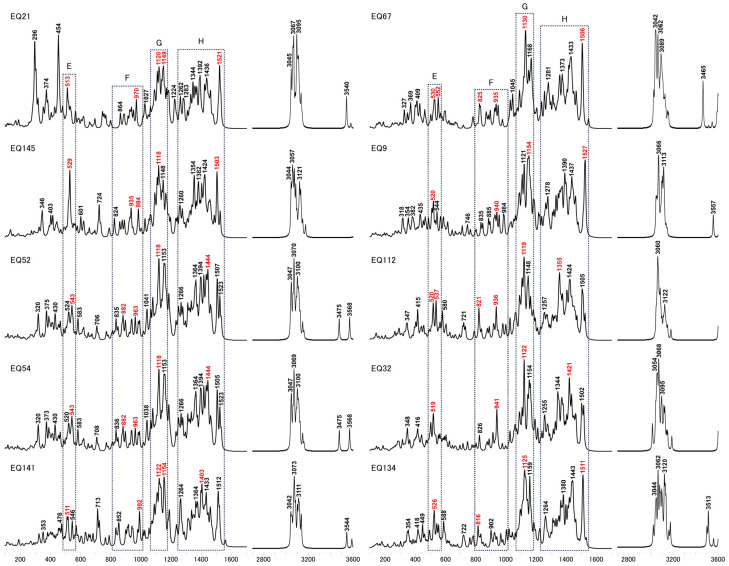
Calculated Raman spectra for explored models, highlighting bands with the highest intensity in four delineated regions*,* E, F, G, and H, denoted in red.

The ten most stable structures derived from the conformational exploration and calculated Raman spectra are shown in Figure 6 and Figure 7. In contrast to their 4Glc1-18 counterparts, these structures exhibited entirely distinct sets of four dihedral angles, as detailed in Table S3. The emergence of coiled conformations prompted notable shifts in intramolecular hydrogen network, involving eight distinct types: O3 (ring 1)⋯H-O6′ (ring 1), O4 (ring 1)⋯H-O6′ (ring 2), O4 (ring 1)⋯H-O6′ (ring 4), O6 (ring 1)⋯H-O6′ (ring 2), O6 (ring 1)⋯H-O6′ (ring 4), O6 (ring 2)⋯H-O6′ (ring 3), O6 (ring 2)⋯H-O6′ (ring 4), and O6 (ring 3)⋯H-O6′ (ring 4). The intramolecular hydrogen bonding patterns observed for each conformation are delineated in Table 3, with the parameters of the dihedral angles provided in Supporting Information Table S3.

For EQ21, ring 3 underwent dehydrogenation, there is an O4⋯H-O6′ hydrogen bond between ring 1 and ring 4, and an O6⋯H-O6′ hydrogen bond between ring 3 and ring 4, although it maintained the α-1,4 linked structure, its stability decreased compared to others; In EQ145, there is a special O3-H⋯O6′ hydrogen bond (153.45°, 1.91 Å) within ring 1, and the O6 of ring 2 forms hydrogen bonds with the O6 of ring 3 and 4, respectively; In EQ52 and EQ54, the O6 of ring 1 forms hydrogen bonds with the O6 of ring 2 and 4, respectively, and there is an O6⋯H-O6′ hydrogen bond between ring 3 and ring 4; In EQ141, there is an O3-H⋯O6′ (165.17°, 1.84 Å) within ring 1 and a relatively strong O4⋯H-O6′ (175.28°, 1.88 Å) hydrogen bond between ring 1 and ring 2, and there are also O6⋯H-O6′ hydrogen bonds between rings 2 and 3, and between rings 3 and 4; In EQ67, there are O6⋯H-O6′ hydrogen bonds between ring 1 and rings 2 and 4, ring 2 and rings 3 and 4, and ring 3 and ring 4; In EQ9, there is a hydrogen bond (166.67°, 1.84 Å) between the O4 of ring 1 and the O6 of ring 4, and a hydrogen bond (163.91°, 1.87 Å) between the O6 of ring 3 and the O6 of ring 4; In EQ112 and EQ32, the O6 of ring 2 forms similar hydrogen bonds with the O6 of ring 3 and 4 (for EQ112: 150.48°, 1.93 Å and 152.49°, 1.88 Å, for EQ32: 151.75°, 1.92 Å and 155.48°, 1.86 Å); In EQ134, there are hydrogen bonds between the O4 of ring 1 and the O6 of ring 4 (168.25°, 1.82 Å), the O6 of ring 2 and the O6 of ring 3 (164.33°, 2.00 Å), and the O6 of ring 3 and the O6 of ring 4 (156.73°, 1.82 Å).

We selected and focused our analysis on four distinct peak regions in the calculated Raman spectra of the coiled glucose models, denoted as E, F, G, and H. Compared to the uncoiled conformation exhibiting the A, B, C, and D regions, the spectral features within each of the newly observed E, F, G, and H regions exhibited discernible alterations. These changes likely arise from forming more intramolecular interactions induced by the conformational changes. Due to dehydrogenation, the EQ21 displayed two prominent bands at 296 and 454 cm^−1^, albeit the four selected regions of interest remained broadly analogous to other structures. In region E, a distinct singular feature emerged at 513 cm^−1^. The spectral region F was characterized by a peak at 970 cm^−1^ and a relatively feeble signal at 864 cm^−1^. Region G showed two peaks of comparable intensities at 1120 and 1149 cm^−1^, while region H featured an intense band at 1521 cm^−1^. Region E of the EQ145 was likewise characterized by a prominent singular band at 529 cm^−1^, exhibiting a higher relative intensity than the features observed in region F. 

In contrast to EQ21, the spectral signature of EQ145 in the region was dominated by an intense peak in region G at 1118 cm^−1^, representing the most pronounced signal across the four regions under investigation. Conversely, in region H, the dominant band for EQ145 was analogous to that of EQ21, located at 1503 cm^−1^. The EQ52 and EQ54 exhibited a comparable distribution of spectral features. In region E, two distinct bands were discernible at 524 and 543 cm^−1^ for EQ52, while EQ54 bands were located at slightly different wavenumbers of 520 and 543 cm^−1^. Region F was characterized by multiple weak peaks. The spectral region G manifested two prominent bands of analogous intensities situated at 1118 and 1153 cm^−1^. The most intense peak across region H occurred at 1444 cm^−1^. The calculated Raman spectra of EQ141 revealed two distinct bands in region E at 511 and 546 cm^−1^, respectively. The region F exhibited the anticipated two prominent peaks at 852 and 992 cm^−1^. In region G, the two bands’ relative intensities at 1122 and 1154 cm^−1^ were comparable. The spectral region H was characterized by the most intense peak across region H, located at 1403 cm^−1^, accompanied by two additional well-defined sharp peaks at 1264 and 1512 cm^−1^. 

The calculated spectrum of EQ67 revealed the presence of two peaks in region E, exhibiting comparable intensities at 530 and 552 cm^−1^. Region F was characterized by two peaks of analogous intensities at 825 and 935 cm^−1^. In region G, a distinct, intense band was observed at 1130 cm^−1^. The spectral region H exhibited a series of four well-defined peaks with gradually increasing intensities, located at 1281, 1373, 1433, and 1506 cm^−1^. The spectral region E of the EQ9 was characterized by the presence of two bands of relatively low intensity at 520 and 544 cm^−1^. The band at 544 cm^−1^ exhibited a comparatively lower intensity and was in close proximity to the prominent peak at 940 cm^−1^ in region F. Region G manifested two distinct bands of substantial intensity at 1121 and 1154 cm^−1^. The spectral region H displayed the most intense peak around 1527 cm^−1^, notably sharp and pronounced compared to the other peaks within the same region. For the case of EQ112, region E was characterized by two well-defined bands at 520 and 537 cm^−1^. Region F exhibited two distinct, prominent peaks at 821 and 936 cm^−1^. In region G, a pronounced intense band was observed at 1119 cm^−1^, showing a significant enhancement in intensity relative to the other regions. Region H revealed three peaks with gradually diminishing intensities at 1355, 1424, and 1505 cm^−1^. In the case of EQ32, region E displayed a peak at 519 cm⁻^1^, which is close to a peak at 941 cm⁻^1^ in region F. However, the peak at 826 cm⁻^1^ in region F exhibited reduced intensity. Region G presented the most intense peak at 1122 cm⁻^1^, comparable to EQ67 and EQ112, while region H showed the most prominent peak at 1421 cm⁻^1^.

In comparison to EQ32, the spectral region E of EQ134 was characterized by a relatively intense band at 526 cm⁻^1^, unlike region F, which did not exhibit the anticipated two distinct prominent peaks. The spectral region G revealed the presence of the most intense band across the four regions at 1125 cm⁻^1^, accompanied by an adjacent band of comparable intensity situated at 1159 cm⁻^1^. Furthermore, region H displayed a pronounced peak at 1511 cm⁻^1^, exhibiting an intensity analogous to the two bands observed in region G.

### 3.2. Band Assignments for 4Glc Structures

The structural investigation into the structural characteristics of 4Glc1-18 and the coiled structures after conformational changes revealed that the rotational behavior of hydroxymethyl groups across the four rings can induce the formation of diverse intramolecular hydrogen bonds, resulting in distinct features within the corresponding vibrational properties. Despite these structural variants exhibiting fluctuations in relative intensity across the two sets of four regions (A, B, C, and D, as well as E, F, G, and H), the positional attributes within similar conformational structures remained notably stable. Specifically, spectral signatures within regions A and B, and corresponding E and F, exhibited relatively simple patterns, facilitating straightforward determination. In contrast, those within regions C and D, and corresponding G and H presented heightened intricacy. 

The comprehensive assignment of calculated Raman spectra presents challenges attributed to vibrational modes’ structural complexity and overlapping nature. In response, our investigation prioritized the examination of vibrational signatures associated with hydroxymethyl groups and α-1,4 glycosidic linkages. Notably, the vibrational modes were classified into stretching (*υ*), bending (*β*), and ring stretching and breathing (ρ), with subscripts indicating the vibration of specific groups occurring at ring *n*. Particular attention was given to discernible bands corresponding to simpler vibrational modes, thereby facilitating the identification of more distinguishable bands applicable to a broader range of cases. The band assignments for the primary vibrations of two spectral parts (100–1700 cm⁻^1^ and 2700–3600 cm⁻^1^) are detailed in Table 4, while the corresponding vibrational animations can be found in the Supporting Information.

### 3.3. Structural Conformations and Calculated Raman Spectra of 2Glc, 6Glc, and 8Glc

**Figure 8:**
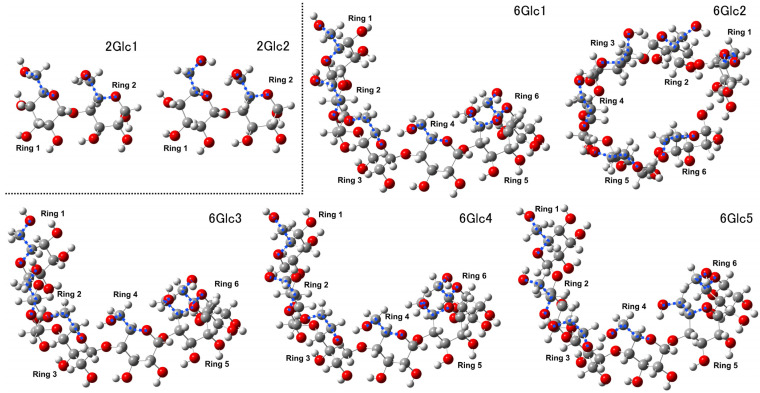
Structural changes observed in 2Glc1-2 and 6Glc1-5 models, highlighting the dihedral angles between specific atoms with blue dashed lines.

The 2Glc structures exhibit a relatively simple conformation, with rotations of the hydroxymethyl group observed in one ring (ring 1). In contrast, the other ring (ring 2) maintains its orientation, as illustrated by the 2Glc1 and 2Glc2 structures in Figure 8. The dihedral angles are provided in Supporting Information Table S4. As the number of amylose units increases to form the 6Glc structures, distortions and curling emerge, and the structures gradually adopt a coiled conformation. Notably, although six amylose units per turn are typically sufficient in starch to form a helical structure, the formation of helical structures is not necessarily observed in our calculated models. This discrepancy may arise from the lack of intramolecular interactions that could restrain the conformational arrangements in these model systems. Further research broadening the perspective to incorporate surrounding molecular movements and environments may be necessary for determining the underlying factors driving the formation of helical structures.

As the number of amylose units increases to eight (8Glc), the structures exhibit more intricate intramolecular hydrogen bonding networks, leading to observations of twisting, curling, or the formation of coiled conformations. The rotations of the hydroxymethyl, represented by the dihedral angles provided in Supporting Information Table S4, facilitate the formation of these weak interactions while simultaneously inducing varying degrees of coiling or partial inversion. To this end, these rotations give rise to diverse coiled or quasi-coiled curved structures, as shown in Figure S1. Notably, the 4Glc structures are intermediate between fully linear and helical conformations. Therefore, investigating the structural properties of 4Glc oligomers offers acceptable computational costs and provides valuable insights into the amylose linkage formation process toward a more complicated system. 

**Figure 9:**
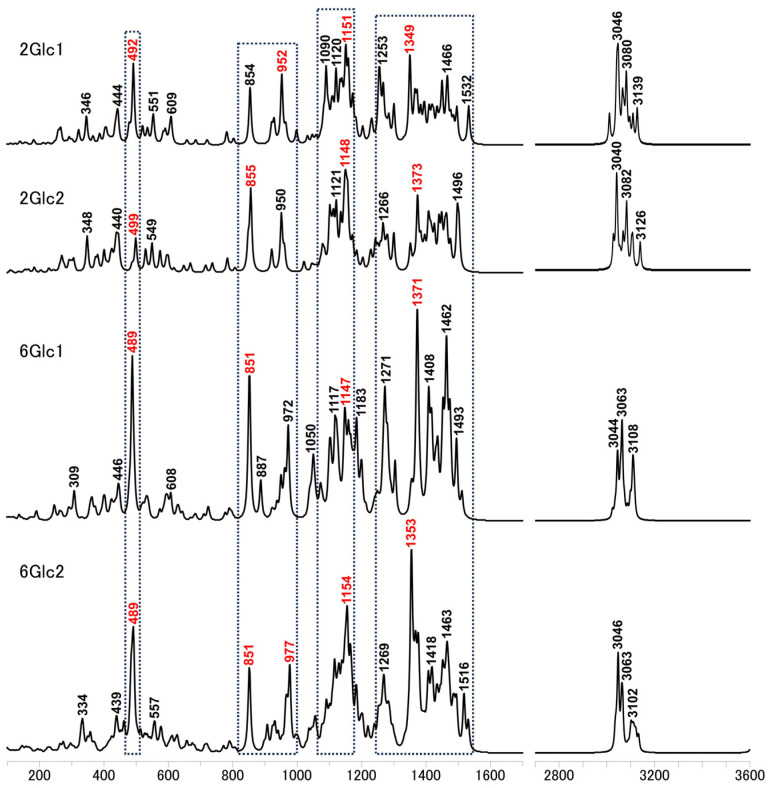
Calculated Raman spectra for representative models of 2Glc and 6Glc, highlighting bands with the highest intensity in comparable regions denoted in red.

Representative calculated Raman spectra for the 2Glc and 6Glc models were presented in Figure 9, with their band assignments of the primary vibrational modes in Table 5, enabling a comparative analysis of their primary vibrational modes. The Raman spectra for selected 8Glc structures, calculated using a different basis set, were provided in Supporting Information Figures S1 and S2. In analyzing the calculated Raman spectra for 2Glc and 6Glc structures, we identified several key differences in the major peaks. For 2Glc1 and 2Glc2, the primary distinctions are the relative intensity of the peak at 492 and 499 cm⁻^1^ and the relative changes in the bands at 854 and 952 cm⁻^1^ and 855 and 950 cm⁻^1^. In both 6Glc1 and 6Glc2, there is a noticeable enhancement of the band at 489 cm⁻^1^. Specifically, for 6Glc1, the band at 851 cm⁻^1^ is significantly stronger than at 972 cm⁻^1^, whereas in 6Glc2, the bands at 851 and 977 cm⁻^1^ are comparable.

A strong peak at 1154 cm⁻^1^ is observed in 6Glc2 but not in 6Glc1. However, 6Glc1 and 6Glc2 exhibit their strongest bands near 1371 and 1353 cm⁻^1^, respectively. This selection aims to provide further insights for various applications of band assignments.

## 4. Conclusions

In this comprehensive quantum chemical exploration, we investigated the structural features and calculated Raman spectra of modeled structures of amylose, encompassing varying units of linked glucose molecules (4Glc, 2Glc, 6Glc, and 8Glc). Our study focused on systematically analyzing 4Glc structures and applied the band feature to other models. The hydroxymethyl group rotation and intramolecular hydrogen bonds were analyzed, revealing that as the number of linked glucose units increases, the linear configuration of linked amylose becomes progressively more intricate, giving rise to curled, cyclic, or helical conformations. Various intramolecular interactions drive these structural transformations, resulting in different vibrational modes of constituent rings in linked glucose structures. The intricate coiling process of amylose is further complicated by the formation of intramolecular hydrogen bonding interactions between various functional groups. These interactions can occur between two hydroxymethyl groups in neighboring rings, an oxygen atom in one ring and a hydroxymethyl group from another ring, or hydroxyl groups from adjacent rings.

The detailed comparison of calculated Raman spectra, emphasizing distinctive vibrational bands, provides critical reference points for reliably assigning vibrational modes across various analytical applications. Despite the intricate structures of amylose molecules, the analyzed vibrational mode can be used as a reference to assign the observed Raman peaks. This enhances our theoretical grasp of carbohydrate structures and broadens research into their spectroscopic properties. By unraveling the spectroscopic fingerprints of linked glucose molecules, this study paves the way for more advanced analysis of complex linked glucose architectures governed by intramolecular hydrogen bond interactions. Importantly, the spectroscopic insights gained herein provide a molecular-level understanding of the formation of biopolymeric structures derived from glucose monomers. The conformational analysis and band assignments lay a foundation for further theoretical and experimental investigations, enabling more accurate interpretation of spectroscopic data for structural analysis of biomolecular systems involving carbohydrate moieties. 

## Figures and Tables

**Table 1:** Intramolecular hydrogen bonds formed by the rotation of hydroxymethyl groups within four rings (4Glc1-4Glc10 structures), showing dihedral angles (in degrees) and hydrogen bond lengths (in Å).

Structure	O5 (Ring 1)⋯H-O6′ (Ring 2)	O5 (Ring 2)⋯H-O6′ (Ring 3)	O5 (Ring 3)⋯H-O6′ (Ring 4)	O6 (Ring 3)⋯H-O6′ (Ring 4)
4Glc1	158.39, 2.06	159.73, 2.10	161.46, 2.09	-
4Glc2	158.21, 2.05	159.93, 2.10	161.29, 2.10	-
4Glc3	158.95, 2.06	160.10, 2.10	-	-
4Glc4	163.07, 2.08	-	-	164.08, 1.89
4Glc5	163.60, 2.11	-	-	163.98, 1.89
4Glc6	-	162.69, 2.09	161.40, 2.09	-
4Glc7	159.34, 1.94	160.02, 2.12	161.23, 2.09	-
4Glc8	-	-	-	158.83, 1.98
4Glc9	160.63, 1.95	-	-	165.88, 1.89
4Glc10	157.96, 1.95	-	154.90, 1.84	158.17, 1.95

**Table 2:** Intramolecular hydrogen bonds formed by the rotation of hydroxymethyl groups within four rings (4Glc11-4Glc18 structures), showing dihedral angles (in degrees) and hydrogen bond lengths (in Å).

Structure	O6 (Ring 1)⋯HOCH2 (Ring 2)	O6 (Ring 2)⋯H-O6′ (Ring 3)	O5 (Ring 3)⋯HOCH2 (Ring 4)	O6 (Ring 3)⋯HOCH2 (Ring 4)
4Glc11	156.42, 2.00	168.22, 1.99	159.78, 2.08	-
4Glc12	156.48, 2.00	168.32, 1.99	159.69, 2.09	
4Glc13	161.70, 1.95	167.75, 1.96	-	-
4Glc14	167.11, 1.87	173.30, 1.85	-	162.54, 1.84
4Glc15	-	158.29, 1.97	162.81, 1.96	-
4Glc16	162.60, 1.92	158.74, 1.89	164.22, 1.95	-
4Glc17	168.32, 1.86	-	-	167.68, 1.88
4Glc18	164.86, 1.86	-	-	161.44, 1.94

**Table 3:** Intramolecular hydrogen bonds formed by the rotation of hydroxymethyl groups within four rings (the explored structures), showing dihedral angles (in degrees) and hydrogen bond lengths (in Å).

Structure	O3 (Ring 1)⋯H-O6′ (Ring 1)	O4 (Ring 1)⋯H-O6′ (Ring 2)	O4 (Ring 1)⋯H-O6′ (Ring 4)	O6 (Ring 1)⋯H-O6′ (Ring 2)	O6 (Ring 1)⋯H-O6′ (Ring 4)	O6 (Ring 2)⋯H-O6′ (Ring 3)	O6 (Ring 2)⋯H-O6′ (Ring 4)	O6 (Ring 3)⋯H-O6′ (Ring 4)
EQ21		-	167.38, 1.82	-	-	-	-	164.81, 1.84
EQ145	153.45, 1.91	-	-	-	-	150.32, 1.93	150.43, 1.90	-
EQ52		-	-	161.65, 1.92	154.06, 1.78	-	-	162.14, 1.86
EQ54		-	-	161.64, 1.92	154.06, 1.78	-	-	162.13, 1.86
EQ141	165.17, 1.84	175.28, 1.88	-	-	-	155.49, 1.93	-	164.93, 1.78
EQ67		-	-	156.21, 1.80	170.57, 1.77	166.54, 1.80	-	154.00, 1.82
EQ9		-	166.67, 1.84	-	-	-	-	163.91, 1.87
EQ112		-	-	-	-	150.48, 1.93	152.49, 1.88	-
EQ32		-	-	-	-	151.75, 1.92	155.48, 1.86	-
EQ134		-	168.25, 1.82	-	-	164.33, 2.00	-	156.73, 1.82

**Table 4:** Raman band assignments for selected 4Glc models with corresponding structure designation following the band wavenumber (cm⁻^1^).

Part	Wavenumber (Structure Name)	Band Assignment (Primary Vibration) ^1^
II	3092 (4Glc14)	υ (4, -CH)
	3046 (4Glc17)	υ (2, -CH)
I	1527 (EQ9)	β (3, 4, -OH), β (1, 2, 3, 4, -CH_2_-OH)
	1462 (4Glc1)	β (2, -OH), β (2, 3, -CH), ρ (2, 3, 4)
	1417 (4Glc8)	υ (2, -C-O-C-), β (1, 2, 3, 4, -CH), β (4, -OH), ρ (3, 4)
	1371 (4Glc1)	β (2, 3, 4, -CH), β (4, -OH), ρ (2, 3, 4)
	1304 (4Glc1)	β (1, -CH), β (1, -OH), β (1, -CH_2_-OH), ρ (1)
	1290 (4Glc13)	β (3, 4, -CH_2_-OH), β (3, 4, -CH), β (4, -OH)
	1280 (4Glc5)	β (1, -CH_2_-OH), β (1, 2, 3, 4, -CH), β (1, 2, 3, 4 -OH)
	1154 (4Glc10)	υ (1, -CH-OH), υ (1, -C-O-C-), β (2, -CH_2_-OH), β (1, 2, 4, -CH), β (1, 2, -OH)
	1123 (4Glc1)	υ (1, 2, 3, 4, -CH-OH), β (1, 2, 3, 4, -CH), β (1, 2, 3, 4 -OH), ρ (1, 2, 3, 4)
	968 (4Glc1)	β (1, 2, 3, 4, -CH_2_-OH), β (1, 2, 3, 4, -CH), β (1, 2, 3, 4, -OH), ρ (1, 2, 3, 4)
	926 (4Glc8)	β (1, 2, 3, 4, -CH_2_-OH), β (1, 2, 3, 4, -CH), β (1, 2, 3, 4, -OH), ρ (1, 2, 3, 4)
	887 (4Glc1)	β (1, -CH_2_-OH), β (1, -CH), ρ (1)
	854 (4Glc1)	β (3, 4, -CH_2_-OH), β (3, 4, -CH), ρ (3, 4)
	529 (EQ145)	β (2, 3, 4, -CH_2_-OH), β (1, 3, -OH), ρ (1, 2, 3)
	490 (4Glc1)	β (1, 2, 3, -CH_2_-OH), β (2, 3, -CH), ρ (1, 2, 3, 4)

^1^ υ—stretching, β—bending, ρ—ring stretching and breathing.

**Table 5:** Raman band assignments for selected 2Glc and 6Glc models, with corresponding structure designation following the band wavenumber (cm⁻^1^).

Part	Wavenumber (Structure Name)	Band Assignment (Primary Vibration) ^1^
II	3046 (6Glc2)	υ (1, 3, 4, -CH), υ (1, 2, 3, -CH_2_-OH)
I	1371 (6Glc1)	υ (2, -CH_2_-OH), β (2, 3, 4, -CH), β (2, 3, 4 -OH), ρ (2, 3, 4)
	1353 (6Glc2)	υ (1, 4, 5, -CH_2_-OH), β (1, 2, 4, 5, 6, -CH), β (1, 4, 5, 6, -OH), ρ (1, 2, 3, 4)
	1154 (6Glc2)	υ (1, 2, 3, 4, 5, 6, -CH_2_-OH), β (1, 2, 3, 4, 5, 6, -CH_2_-OH), β (1, 2, 3, 4, 5, 6, -CH), β (1, 2, 3, 4, 5, 6, -OH), ρ (1, 2, 3, 4, 5, 6)
	977 (6Glc2)	υ (2, 3, 4, 5, 6, -CH_2_-OH), β (1, 2, 3, 4, 5, 6, -CH), β (1, 2, 3, 4, 5, 6, -OH), ρ (1, 2, 3, 4, 5, 6)
	851 (6Glc1)	β (2, 3, 4, 5, 6, -CH_2_-OH), β (2, 3, 4, 6, -CH), ρ (2, 3, 4, 6)
	489 (6Glc1)	β (2, 3, 4, 5, 6, -CH_2_-OH), ρ (1, 2, 3, 4, 5, 6)

^1^ υ—stretching, β—bending, ρ—ring stretching and breathing.

## Data Availability

Data are contained within the article and Supplementary Materials.

## Supplementary Materials

The following supporting information can be downloaded at: https://www.mdpi.com/article/10.3390/molecules29122842/s1, Table S1: Dihedral angles (in degrees) describing the orientations of hydroxymethyl groups in each glucose ring for the 4Glc1-4Glc10 structural models; Table S2: Dihedral angles (in degrees) describing the orientations of hydroxymethyl groups in each glucose ring for the 4Glc11-4Glc18 structural models; Table S3: Dihedral angles (in degrees) describing the orientations of hydroxymethyl groups in each glucose ring for the explored structures; Table S4: Dihedral angles (in degrees) describing the orientations of hydroxymethyl groups in each glucose ring for the 2Glc1-2 and 6Glc1-5 structural models; Table S5: Dihedral angles (in degrees) describing the orientations of hydroxymethyl groups in each glucose ring for the 8Glc1-10 structural models; Figure S1: Structural changes observed in 8Glc1-10 models, highlighting the dihedral angles between specific atoms with blue dashed lines; Figure S2. Calculated Raman spectra for 8Glc1-10 models, with the most intense bands in four selected regions highlighted in red (calculated at the CAM-B3LYP/6-31G(d) level of theory); Appendix SA: Detailed diagrams of hydrogen bonding formations in the linear models; Vibrational animations can be found in Band_Assignment.rar.
