# Supplementary material for: Quantum Chemical Investigation into the Structural Analysis and Calculated Raman Spectra of Amylose Modeled with Linked Glucose Molecules
Source: Molecules. 2024 Jun 14;29(12):2842. doi: 10.3390/molecules29122842 (PMC11206574; doi:10.3390/molecules29122842)

Quantum Chemical Investigation into the Structural Analysis and Calculated Raman Spectra of Amylose Modeled with Linked Glucose Molecules

Dapeng Zhang,[†] *Naoki Kishimoto*[*†]

[†] Department of Chemistry, Graduate School of Science, Tohoku University, 6-3, Aoba, Aramaki, Aoba-ku, Sendai 980-8578, Japan

Corresponding Author

***E-mail**: kishimoto@tohoku.ac.jp

Supporting Information

Table of Contents

Table S1. Dihedral angles for 4Glc1-4Glc10 models ……………………......................**S3**

Table S2. Dihedral angles for 4Glc11-4Glc18 models ……………………......................**S4**

Table S3. Dihedral angles for explored structures ……………………............................**S5**

Table S4. Dihedral angles for 2Glc1-2 and 6Glc1-5 models ……...............................**S6**

Table S5. Dihedral angles for 8Glc1-10 models……..**S7**

Figure S1. Structures of the 8Glc1-10 models ……………………...........................……**S8**

Figure S2. Calculated Raman spectra for 8Glc1-10 models………………………......……**S9**

Appendix SA: Detailed diagrams of hydrogen bonding formations in the linear models

Table S1. Dihedral angles (in degrees) describing the orientations of hydroxymethyl groups in each glucose ring for the 4Glc1-4Glc10 structural models.

Structure	Relative Energy (CAM-B3LYP, kJ/mol, 298.15 K)	Change in dihedral angle 1	Change in dihedral angle 2	Change in dihedral angle 3	Change in dihedral angle 4
4Glc1	18.09	-75.76 to -66.70	-75.76 to -81.12	-26.85 to -82.00	-26.85 to -81.49
4Glc2	26.69	-26.85 to -66.67	-26.85 to -81.09	-26.85 to -81.90	-26.85 to -82.29
4Glc3	34.98	-78.73 to -66.75	-78.73 to -81.11	-15.24 to -82.61	-15.24 to 78.30
4Glc4	17.36	-15.24 to -82.18	-15.24 to -82.53	-15.24 to 75.68	-15.24 to 147.89
4Glc5	8.95	-78.73 to -177.38	-78.73 to -82.38	81.29 to 75.34	81.29 to 148.06
4Glc6	18.68	153.87 to -176.97	153.87 to 152.41	-74.76 to -82.10	-74.76 to -82.28
4Glc7	0 (-2519.28 Hartree)	153.87 to -174.49	153.87 to 168.55	-26.85 to -82.40	-26.85 to -81.51
4Glc8	13.89	153.87 to -177.03	153.87 to 156.43	81.29 to 55.96	81.29 to 151.35
4Glc9	8.43	153.87 to -174.92	153.87 to 168.24	-15.24 to 72.53	-15.24 to 148.49
4Glc10	3.83	153.87 to -173.37	153.87 to 170.75	153.87 to 94.97	153.87 to 171.18

Table S2. Dihedral angles (in degrees) describing the orientations of hydroxymethyl groups in each glucose ring for the 4Glc11-4Glc18 structural models.

Structure	Relative Energy (CAM-B3LYP, kJ/mol, 298.15 K)	Change in dihedral angle 1	Change in dihedral angle 2	Change in dihedral angle 3	Change in dihedral angle 4
4Glc11	0 (-2519.28 Hartree)	20.32 to 73.05	20.32 to 88.32	-74.76 to -75.63	-74.76 to -81.33
4Glc12	8.73	81.29 to 73.07	81.29 to 88.44	-26.85 to -75.54	-26.85 to -82.14
4Glc13	20.83	81.29 to 72.11	81.29 to 87.68	-15.24 to -75.71	-15.24 to 77.92
4Glc14	0.35	81.29 to 71.03	81.29 to 96.85	-15.24 to 93.53	-15.24 to 145.06
4Glc15	20.26	-15.24 to 70.96	-15.24 to 57.27	153.87 to 156.62	153.87 to 166.08
4Glc16	10.96	81.29 to 71.85	81.29 to 96.93	153.87 to 152.57	153.87 to 164.83
4Glc17	11.65	-15.24 to 71.04	-15.24 to 139.65	81.29 to 73.00	81.29 to 148.00
4Glc18	19.42	81.29 to 69.87	81.29 to 151.75	81.29 to 64.18	81.29 to 141.41

Table S3. Dihedral angles (in degrees) describing the orientations of hydroxymethyl groups in each glucose ring for the explored structures.

Structure	Relative Energy (CAM-B3LYP, kJ/mol, 298.15 K)	Dihedral angle 1	Dihedral angle 2	Dihedral angle 3	Dihedral angle 4
EQ21	170.49	163.68	105.82	140.77	95.88
EQ145	90.41	-60.81	101.02	88.46	170.61
EQ52	24.89	100.66	95.03	146.86	113.25
EQ54	34.76	100.72	95.06	146.80	113.28
EQ141	81.90	-55.91	134.33	137.58	106.97
EQ67	0 (-2519.22 Hartree)	74.04	109.01	87.62	136.74
EQ9	46.75	163.79	105.30	144.62	95.88
EQ112	108.19	168.72	101.59	87.20	171.53
EQ32	68.90	-179.05	103.52	86.23	172.67
EQ134	85.74	-166.46	143.92	107.53	139.09

Table S4. Dihedral angles (in degrees) describing the orientations of hydroxymethyl groups in each glucose ring for the 2Glc1-2 and 6Glc1-5 structural models.

Structure	Relative Energy (CAM-B3LYP, kJ/mol, 298.15 K)	Dihedral angle 1	Dihedral angle 2	Dihedral angle 3	Dihedral angle 4	Dihedral angle 5	Dihedral angle 6	Dihedral angle 7	Dihedral angle 8
2Glc1	0 (-1297.85 Hartree)	-177.07	-81.18				-		
2Glc2	7.59	78.71	-81.48						
6Glc1	42.44	-66.74	-81.06	-81.96	-82.22	-82.09	-82.14		
6Glc2	0 (-3740.71 Hartree)	-179.37	-84.98	-86.24	-151.57	-155.70	-82.89		
6Glc3	34.72	-177.20	150.65	-82.19	-82.10	-82.03	-82.22	-	
6Glc4	31.82	-81.96	-81.97	-82.00	-82.13	-82.00	-82.14		
6Glc5	31.30	-81.64	-81.93	-82.02	-81.98	168.28	-82.71		

Table S5. Dihedral angles (in degrees) describing the orientations of hydroxymethyl groups in each glucose ring for the 8Glc1-10 structural models.

Structure	Relative Energy (CAM-B3LYP, kJ/mol, 298.15 K)	Dihedral angle 1	Dihedral angle 2	Dihedral angle 3	Dihedral angle 4	Dihedral angle 5	Dihedral angle 6	Dihedral angle 7	Dihedral angle 8
8Glc1	0 (-4960.47 Hartree)	-64.35	-84.18	-83.37	-87.57	-90.80	-90.40	-86.06	-71.64
8Glc2	21.79	69.36	146.33	67.31	159.27	70.86	97.85	95.17	144.23
8Glc3	151.90	-177.24	170.80	170.12	176.01	169.90	-158.02	99.89	173.19
8Glc4	64.50	72.57	88.23	-77.01	-82.74	-82.82	-84.17	53.98	154.60
8Glc5	23.15	72.76	88.85	-82.98	-97.31	-94.45	-89.57	77.95	105.57
8Glc6	140.51	-178.04	158.70	-82.85	-163.02	-157.97	-85.59	165.15	171.45
8Glc7	3.53	76.32	94.83	-75.44	-84.98	71.34	99.58	97.85	148.80
8Glc8	30.21	-177.57	167.08	69.44	132.16	74.35	101.05	150.21	162.60
8Glc9	151.00	168.86	154.18	96.86	71.87	148.56	67.43	169.00	-177.34
8Glc10	8.94	70.77	154.39	67.79	152.49	72.96	-74.31	63.03	63.03

Figure S1. Structural changes observed in 8Glc1-10 models, highlighting the dihedral angles between specific atoms with blue dashed lines.

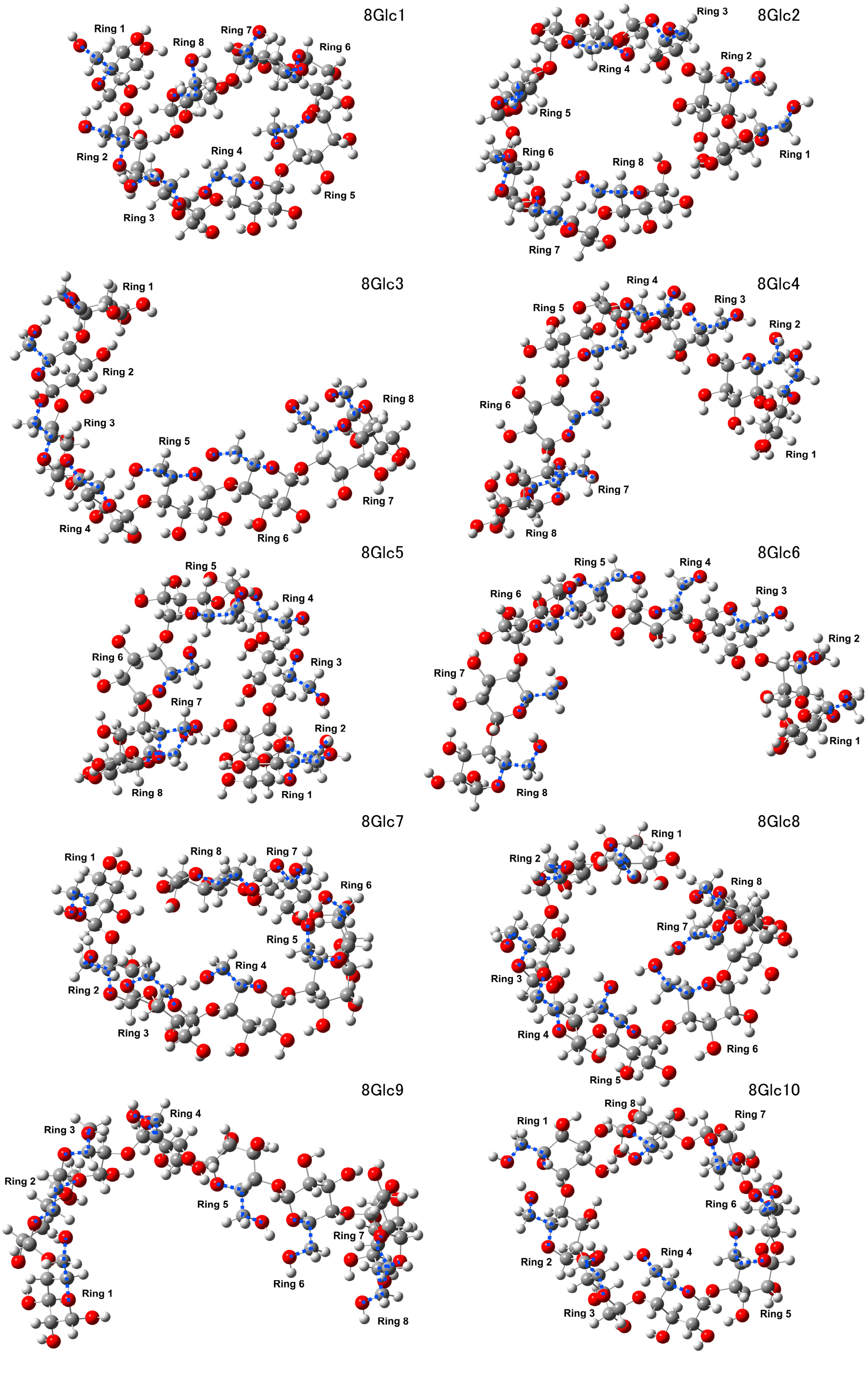

Figure S2. Calculated Raman spectra for 8Glc1-10 models, with the most intense bands in four selected regions highlighted in red (calculated at the CAM-B3LYP/6-31G(d) level of theory).

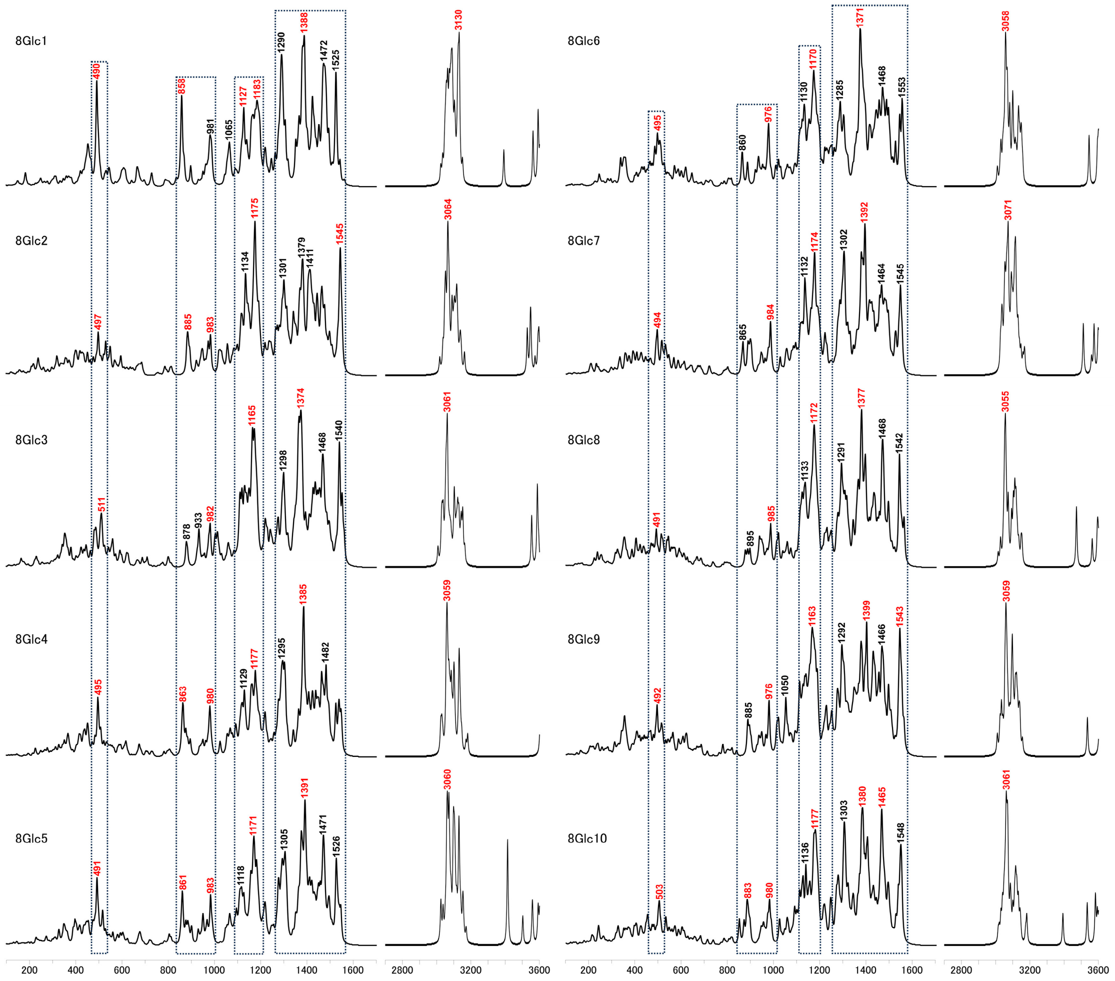

Appendix SA: Detailed diagrams of hydrogen bonding formations in the linear models

4Glc1

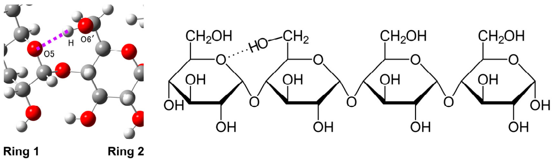

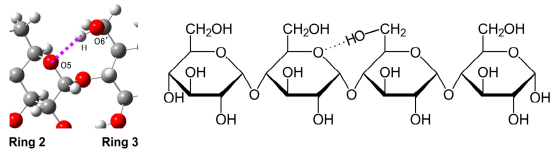

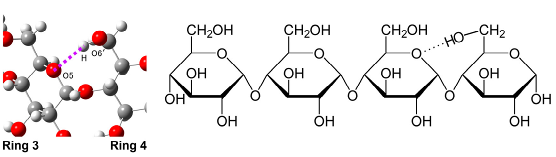

4Glc2

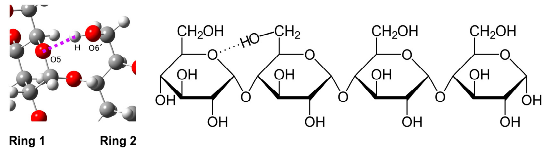

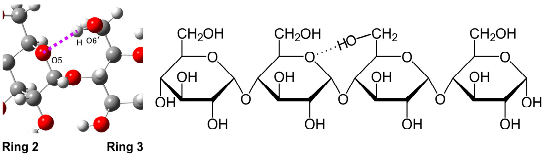

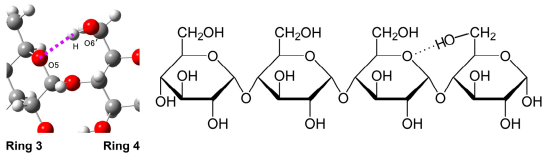

4Glc3

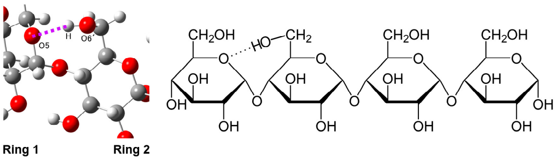

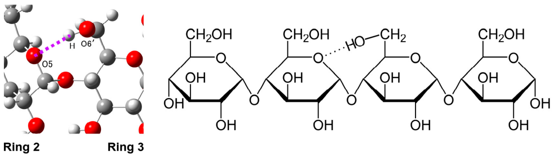

4Glc4

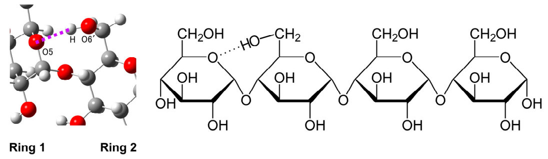

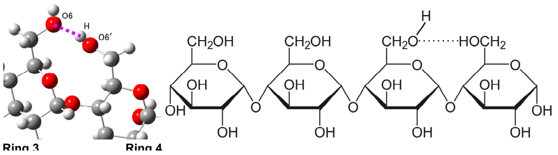

4Glc5

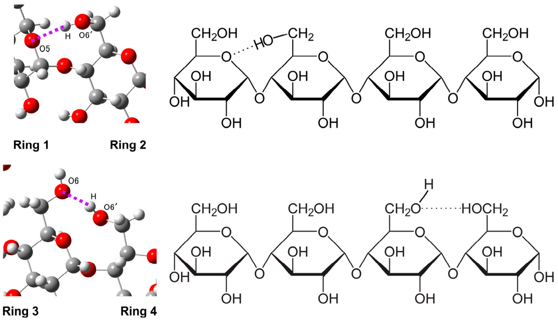

4Glc6

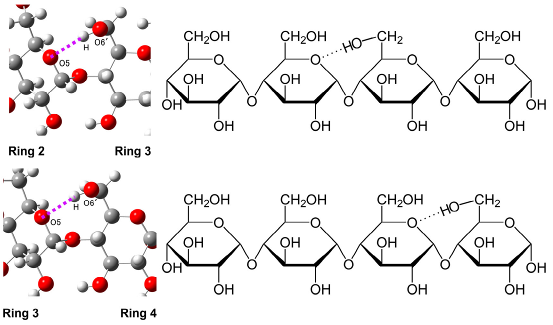

4Glc7

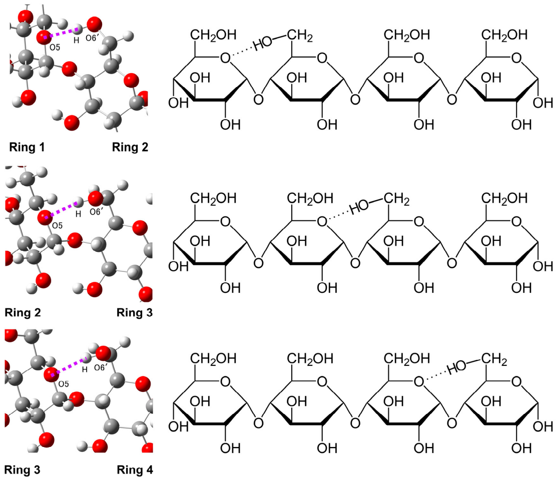

4Glc8

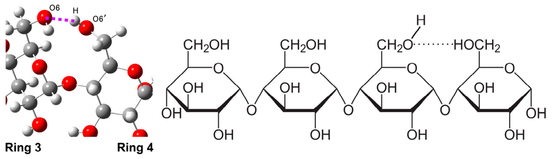

4Glc9

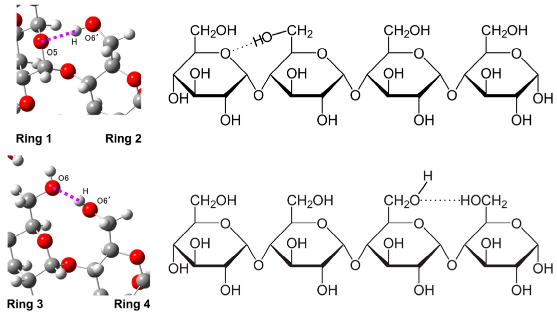

4Glc10

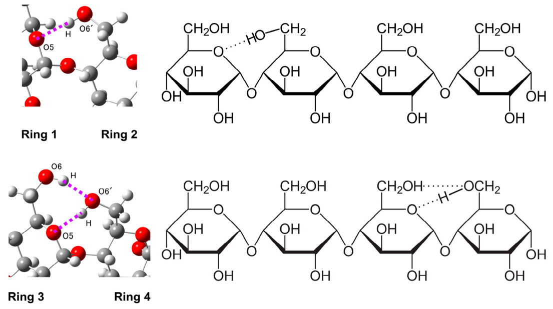

4Glc11

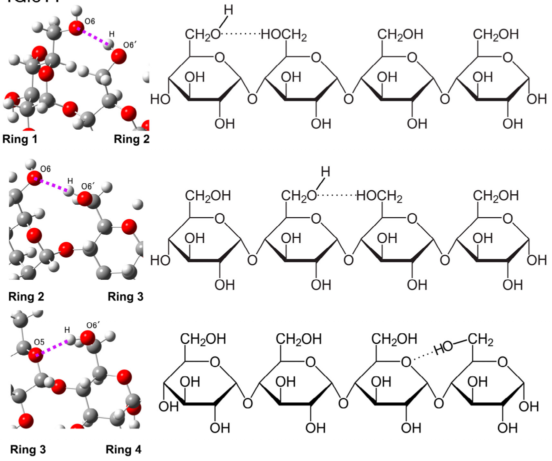

4Glc12

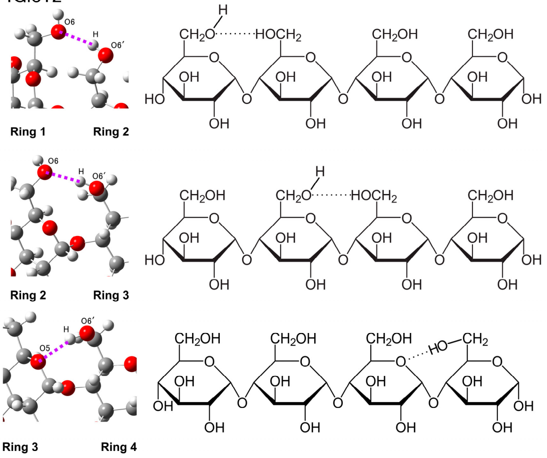

4Glc13

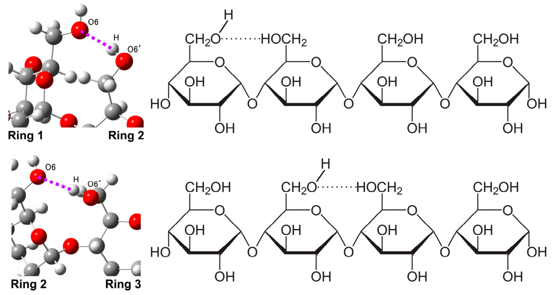

4Glc14

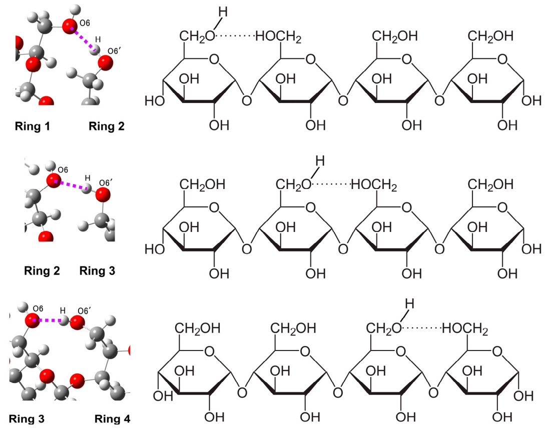

4Glc15

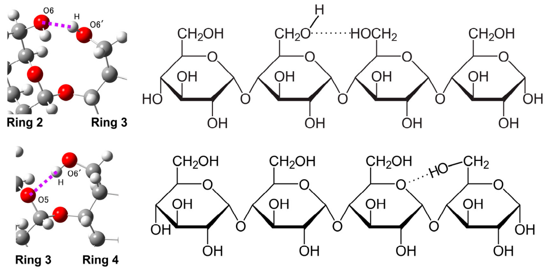

4Glc16

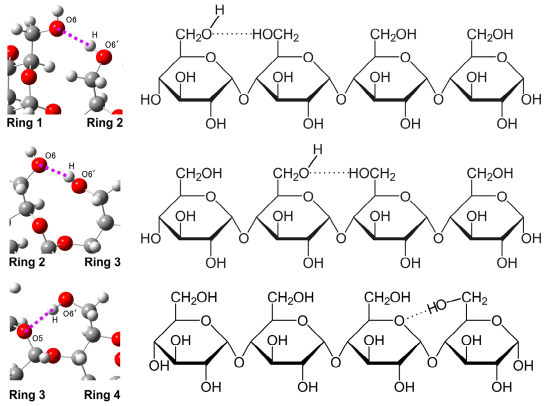

4Glc17

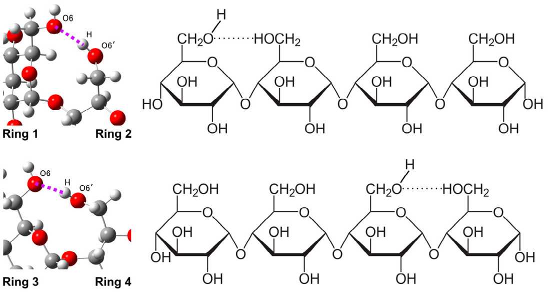

4Glc18

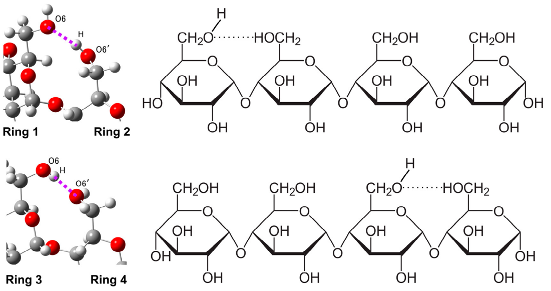

Supplement: Supplementary file 1 [file molecules-29-02842-s001.zip › molecules-3056918-supplementary.pdf]
